# 气相色谱-质谱法测定化妆品中13种防晒剂

**DOI:** 10.3724/SP.J.1123.2020.11003

**Published:** 2021-05-08

**Authors:** Wen LÜ, Hongying LI, Jie LIU, Wei HAN, Wei HUANG

**Affiliations:** 湖北省药品监督检验研究院, 湖北 武汉 430075; Hubei Institute for Drug Control, Wuhan 430075, China; 湖北省药品监督检验研究院, 湖北 武汉 430075; Hubei Institute for Drug Control, Wuhan 430075, China; 湖北省药品监督检验研究院, 湖北 武汉 430075; Hubei Institute for Drug Control, Wuhan 430075, China; 湖北省药品监督检验研究院, 湖北 武汉 430075; Hubei Institute for Drug Control, Wuhan 430075, China; 湖北省药品监督检验研究院, 湖北 武汉 430075; Hubei Institute for Drug Control, Wuhan 430075, China

**Keywords:** 气相色谱-质谱法, 防晒剂, 化妆品, gas chromatography-mass spectrometry (GC-MS), sunscreen agents, cosmetics

## Abstract

防晒剂广泛应用于化妆品中,是目前化妆品监管的重点。建立了同时测定化妆品中13种防晒剂的气相色谱-质谱(GC-MS)方法。化妆品样品经二氯甲烷提取后,涡旋超声后稀释。采用程序升温模式,经HP-5ms毛细管色谱柱(30 m×250 μm×0.25 μm)在30 min内对13种防晒剂实现分离,经电子轰击(EI)源电离后采用选择离子监测模式(SIM)扫描测定,外标法定量。比较了6种常用有机溶剂的基质效应和平均回收率,二氯甲烷的基质效应弱,平均回收率较高。13种防晒剂在相应的线性范围内线性关系良好,相关系数均大于0.998,检出限(LOD, *S/N*=3)为0.04~0.63 mg/g,定量限(LOQ, *S/N*=10)为0.12~2.10 mg/g。实验选取了两种基质,在3个水平下验证方法的回收率和精密度,13种防晒剂在霜类基质中的加标回收率为88.7%~103.6%,相对标准偏差(RSD, *n*=6)为1.7%~4.9%,在乳类基质中的加标回收率为88.4%~102.3%, RSD(*n*=6)为1.2%~3.9%。美白类化妆品常添加防晒剂成分,为监管盲区,采用该方法检测了5批含有防晒剂的美白类化妆品,其所含5种防晒剂的含量为0.8%~5.2%,符合相关要求。该方法操作简单,灵敏度高,回收率好,测定的13种防晒剂均为我国《化妆品安全技术规范》2015版规定的常用的限用组分,可以用于各类化妆品中13种防晒剂的定性定量测定,为市场监管和实验室检测提供新的技术支持。

## Abstract (en)

Sunscreens can be categorized as physical and chemical types. Chemical sunscreens are widely used in cosmetics, and hence, their concentration in the desired products should be strictly monitored. Gas chromatography-mass spectrometry (GC-MS) is widely used for the analysis of cosmetics as it does not require organic mobile phases and allows for accurate qualitative and quantitative analyses. In this study, a method based on GC-MS was established for the determination of 13 sunscreen agents in cosmetics: ethylhexyl salicylate, homosalate, 4-methylbenzylidene camphor, ethylhexyl dimethyl para-aminobenzoic acid, ethylhexyl methoxycinnamate, octocrylene, butyl methoxydibenzoylmethane, diethylamino hydroxybenzoyl hexyl benzoate, 3-benzylidene camphor, benzophenone-3, camphor benzalkonium methosulfate, drometrizole trisiloxane, and isopentyl-4-methoxycinnamate. Accordingly, 0.5 g of the cosmetic product was dissolved in dichloromethane in a 50 mL volumetric flask and extracted ultrasonically for 15 min. Then, 1.0 mL of the extracting solution was withdrawn and diluted to 50.0 mL with dichloromethane. The mixture was filtered through a 0.22-μm membrane. A 1 μL aliquot of the dichloromethane solution was introduced onto the HP-5ms chromatographic column (30 m×250 μm×0.25 μm). The 13 components were separated under programed temperature elevation in the interval from 150 ℃ to 290 ℃. These components could be analyzed within 30 min after being ionized by the EI source, and their determination was achieved in selected ion monitoring (SIM) mode. The external standard method was employed for quantitative determination. Linear equations, linear correlation coefficients, and linear ranges were obtained by analyzing a series of mixed standard working solutions. The limits of detection (LODs, *S/N*=3) and limits of quantification (LOQs, *S/N*=10) of the 13 sunscreen agents were determined. The matrix effect and average recoveries of the 13 agents with six extraction solvents (dichloromethane, tetrahydrofuran, methanol, acetonitrile, *n*-hexane, and acetone) were compared. Among these, dichloromethane showed a weak matrix effect and high average recovery. The matrix effect of dichloromethane was 90.1%-100.5%, and the average recovery was 96.3%. All the 13 sunscreen agents showed good linearity in their corresponding ranges. The correlation coefficients (*r* ^2^) were higher than 0.998. The LODs and LOQs were in the ranges of 0.04 to 0.63 mg/g and 0.12 to 2.10 mg/g, respectively. Two types of cosmetics were selected to verify the accuracy and precision of the method at three levels. The average spiked recoveries of the 13 sunscreen agents in cream and lotion were 88.7%-103.6%, and 88.4%-102.3%, respectively; the corresponding relative standard deviations (RSDs, *n*=6) were 1.7%-4.9% and 1.2%-3.9%. Whitening cosmetics are frequently added with sunscreen agents, which is a regulatory blind spot. Five batches of skin whitening products containing sunscreen agents were detected using this method. The contents of five sunscreen agents in skin whitening cosmetics ranged from 0.8% to 5.2%, which were lower than the relevant limits in China. Owing to its advantages of simple operation, high sensitivity, and good recovery, the proposed method is suitable for the qualitative and quantitative determination of 13 sunscreen agents in cosmetics. This method provides technical support for market supervision and laboratory testing.

随着人们生活水平的提高,防晒意识逐渐增强,防晒类产品的使用也越来越频繁^[1]^。防晒剂可以分为物理防晒剂和化学防晒剂,化学防晒剂由于防晒效果好、种类繁多而广泛用于各类化妆品中^[2,3,4]^。但长期大量使用化学防晒剂会对人体皮肤造成损害,引起过敏、皮炎等不良反应,因此世界各国对防晒剂的使用限量有明确的要求^[5,6]^。我国《化妆品安全技术规范》2015年版规定了25种准用化学防晒剂和2种物理防晒剂的使用限度^[7]^。目前,化学防晒剂的检测方法主要有高效液相色谱法^[8,9,10,11]^、液相色谱-质谱联用法^[12,13]^、气相色谱法^[14]^、气相色谱-质谱法^[15,16,17]^等。其中气相色谱-质谱法由于不使用有机流动相、定性定量准确等优点而广泛用于化妆品检验检测^[18]^。目前关于气相色谱-质谱法检测化妆品中防晒剂的研究较少,检测对象不够全面^[19,20,21,22]^。本文针对我国《化妆品安全技术规范》2015年版规定的25种准用化学防晒剂中的13种成分,建立气相色谱-质谱分析方法,进一步完善相关检测方法。

## 1 实验部分

### 1.1 仪器、试剂与材料

Agilent 7890A-7000B气相色谱-三重四极杆质谱仪(美国安捷伦公司); HMV-50A型涡旋振荡器(天津恒奥公司); BRANSON-8800型超声波清洗机(上海必能信公司)。

13种防晒剂:水杨酸乙基己酯(ethylhexyl salicylate, ES,纯度99.40%)、胡莫柳酯(homosalate, HMS,纯度99.6%)、4-甲基苄亚基樟脑(4-methylbenzylidene camphor, 4-MBC,纯度99.09%)、二甲基对氨基苯甲酸乙基己酯(ethylhexyl dimethyl para- aminobenzoic acid, ED-PABA,纯度98.63%)、甲氧基肉桂酸乙基己酯(ethylhexyl methoxycinnamate, EHMC,纯度99.39%)、奥克立林(octocrylene, OC,纯度98.7%)、丁基甲氧基二苯甲酰基甲烷(butyl methoxydibenzoylmethane, BMDBM,纯度99.8%)、二乙氨羟苯甲酰基苯甲酸己酯(diethylamino hydroxybenzoyl hexyl benzoate, DHHB,纯度99.43%)(德国Dr. Ehrenstorfer公司); 3-亚苄基樟脑(3-benzylidene camphor, 3-BC,纯度96%)(加拿大TRC公司);二苯酮-3(benzophenone-3, BP-3,纯度99.24%)(美国Stanford Chemicals公司);樟脑苯扎铵甲基硫酸盐(camphor benzalkonium methosulfate, CBM,纯度99.2%)(美国Sigma公司);甲酚曲唑三硅氧烷(drometrizole trisiloxane, DT,纯度99.4%)、对甲氧基肉桂酸异戊酯(isopentyl-4-methoxycinnamate, IMC,纯度98.9%)(德国USP公司)。

二氯甲烷(dichloromethane, DCM)、四氢呋喃(tetrahydrofuran, THF)、甲醇(methanol, MT)、乙腈(acetonitrile, ACN)、正己烷(*n*-hexane, HA)、丙酮(acetone, AT),色谱纯,德国默克公司。

### 1.2 标准溶液制备

标准储备溶液:分别称取13种防晒剂标准品0.05 g于10 mL棕色容量瓶中,用二氯甲烷定容至刻度配制成5 g/L的标准储备溶液。

混合标准溶液:分别移取丁基甲氧基二苯甲酰基甲烷和二乙氨羟苯甲酰基苯甲酸己酯标准储备溶液各5 mL,樟脑苯扎铵甲基硫酸盐、奥克立林和甲酚曲唑三硅氧烷标准储备溶液各2.5 mL,其他标准储备溶液各0.5 mL于50 mL容量瓶中,用二氯甲烷定容至刻度配制成13种防晒剂的混合标准溶液。

### 1.3 样品前处理

准确称取0.5 g(精确到0.1 mg)化妆品样品于50 mL容量瓶中,用二氯甲烷定容至刻度,涡旋振荡30 s,超声萃取15 min,取该溶液1 mL,再用二氯甲烷稀释至50 mL,经0.22 μm有机系微孔滤膜过滤,待测。

### 1.4 气相色谱-质谱条件

色谱柱:HP-5ms毛细管色谱柱(30 m×250 μm×0.25 μm);载气:高纯氮气;柱流速:1.0 mL/min;进样量:1 μL;进样方式:分流进样,分流比为10∶1;进样口温度:260 ℃。柱温采用程序化升温:初始温度150 ℃,以5 ℃/min升温至290 ℃,保持5 min。

离子源为EI源;离子源温度为230 ℃;四极杆温度:150 ℃;电子能量:70 eV;采用选择离子监测模式(SIM)扫描。

## 2 结果与讨论

### 2.1 GC-MS条件的选择

本实验采用HP-5ms非极性毛细管色谱柱对13种防晒剂进行分离,通过全扫描结合NIST谱库检索,选择丰度较高、干扰较低、重现性好的3个特征离子作为定性离子,其中丰度最高的一个作为定量离子,然后采用SIM模式进行测定,结果见表1。

**表 1:** 13种防晒剂的保留时间、定量和定性离子

No.	Compound	CAS No.	Retention time/min	Quantitative ion (*m/z*)	Qualitative ions (*m/z*)
1	ES	118-60-5	8.72	120	138, 121
2	HMS	118-56-9	10.09	138	69, 109
3	3-BC	36861-47-9	10.94	128	240, 129
4	BP-3	22071-24-5	12.52	227	151, 228
5	IMC	71617-10-2	12.58	178	161, 133
6	4-MBC	36861-47-9	13.01	254	128, 115
7	ED-PABA	58817-05-03	16.44	165	148, 164
8	EHMC	5466-77-3	17.22	178	161, 133
9	CBM	52793-97-2	20.26	240	283, 134
10	OC	6197-30-4	22.64	204	232, 248
11	BMDBM	70356-09-1	24.25	310	135, 295
12	DT	155633-54-8	25.79	221	73, 369
13	DHHB	302776-68-7	28.77	382	397, 383

ES: ethylhexyl salicylate; HMS: homosalate; 3-BC: 3-benzylidene camphor; BP-3: benzophenone-3; IMC: isopentyl-4-methoxycinnamate; 4-MBC: 4-methylbenzylidene camphor; ED-PABA: ethylhexyl dimethyl para-aminobenzoic acid; EHMC: ethylhexyl methoxycinnamate; CBM: camphor benzalkonium methosulfate; OC: octocrylene; BMDBM: butyl methoxydibenzoylmethane; DT: drometrizole trisiloxane; DHHB: diethylamino hydroxybenzoyl hexyl benzoate.

采用SIM模式扫描得到的13种防晒剂的总离子流色谱图(TIC)见图1,可以看出,13种防晒剂在30 min内得到较好的分离,峰形尖锐对称。

**图 1:**
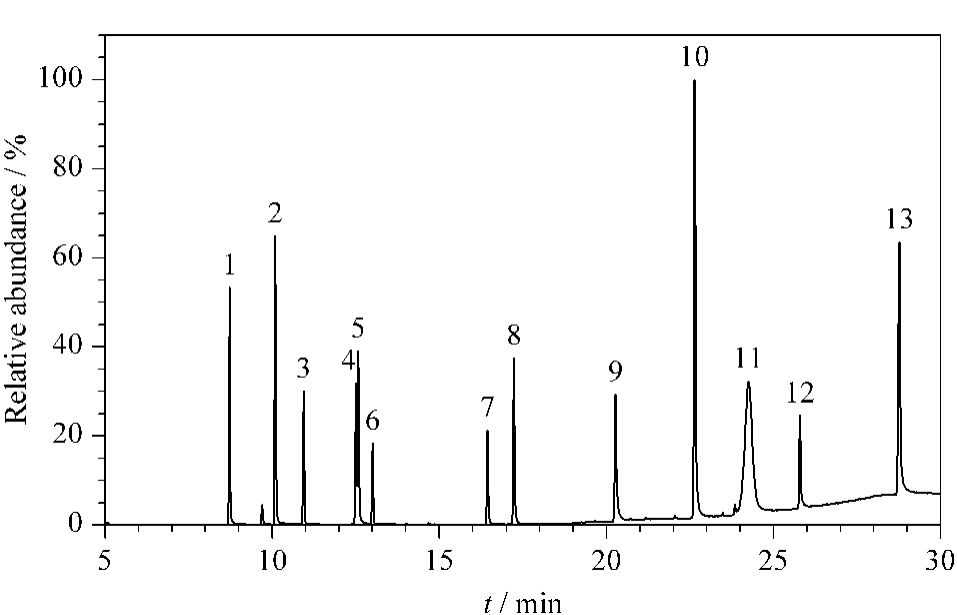
13种防晒剂的总离子流图

### 2.2 萃取溶剂的选择与基质效应

本文试验了气相色谱常用的不同极性的6种有机溶剂来进行萃取,考察了6种溶剂对13种防晒剂的平均回收率的影响。同时,由于化妆品基质复杂,其基质效应(matrix effect, ME)直接影响结果的灵敏度。取空白样品按10 mg/g的水平添加13种防晒剂,用6种不同溶剂按1.3节方法处理后配制基质标准溶液(A),同时以对应纯溶剂配制相同浓度的标准溶液(B),按公式ME=(A溶液的响应值/B溶剂中相应目标物的响应值)×100%计算,进行基质效应评价。平均回收率和基质效应结果见表2。由表2可知,二氯甲烷、丙酮和四氢呋喃的平均回收率最大,其中二氯甲烷的平均回收率为96.3%。13种防晒剂的ME各不相同,其中二氯甲烷的ME为90.1%~100.5%,表明其基质效应弱,特异性良好,而其他溶剂的ME值都有小于90%的情况,对某些组分抑制效应明显;同时考虑到丙酮为易制毒类试剂,四氢呋喃对仪器有一定的腐蚀性,故选择二氯甲烷作为萃取溶剂。

**表 2:** 13种防晒剂在不同溶剂中的基质效应和平均回收率

Solvent	MEs/%													AR/%
	1	2	3	4	5	6	7	8	9	10	11	12	13	
DCM	94.1	96.1	93.7	90.1	96.0	99.5	93.4	92.3	93.7	93.2	98.5	99.7	100.5	96.3
MT	82.4	88.8	87.3	87.4	87.8	93.9	76.2	85.5	97.5	83.2	93.3	89.1	94.0	89.2
ACN	86.0	90.7	88.4	89.6	88.9	92.8	77.2	100.4	92.7	90.2	93.5	96.7	95.7	90.1
AT	92.5	92.8	93.5	94.4	94.5	93.4	89.9	91.3	98.5	96.1	98.1	92.2	92.8	92.8
HA	68.5	67.3	68.8	63.3	70.6	75.3	77.0	67.2	62.4	67.8	74.9	66.9	71.4	71.4
THF	90.1	101.2	94.9	92.7	96.6	100.3	85.2	85.0	83.4	89.7	99.6	93.6	100.2	95.5

* For Nos.1-13, see Table 1. DCM: dichloromethane; MT: methanol; ACN: acetonitrile; AT: acetone; HA: *n*-hexane; THF: tetrahydrofuran.

### 2.3 线性关系与检出限

分别取混合标准溶液0.05、0.1、0.2、0.4、0.8和1.5 mL,用二氯甲烷定容至10 mL容量瓶中,得到标准系列溶液,依次测定。以13种防晒剂定量离子的峰面积(*y*)为纵坐标,进样浓度(*x*)为横坐标,绘制标准曲线,线性参数见表3。由表3可以看出,13种目标物在相应的线性范围内线性关系良好,相关系数大于0.998。取不含目标物的防晒化妆品,添加一定低浓度的13种防晒剂混合标准溶液,按样品提取方法处理后测定,以3倍信噪比计算检出限,10倍信噪比计算定量限,13种防晒剂的检出限为0.04~0.63 mg/g,定量限为0.12~2.10 mg/g,满足实验分析要求。

**表 3:** 13种防晒剂的线性方程、线性范围和检出限

Compound	Linear equation	*r* ^2^	Linear range/(mg/L)		LOD/(mg/g)	LOQ/(mg/g)
ES	*y*=14236.13*x*-275.39	0.9982	0.25-	7.5	0.05	0.17
HMS	*y*=8027.37*x*-483.33	0.9983	0.25-	7.5	0.07	0.23
3-BC	*y*=7581.82*x*-1287.27	0.9996	0.25-	7.5	0.06	0.20
BP-3	*y*=10902.47*x*-339.78	0.9985	0.25-	7.5	0.06	0.20
IMC	*y*=18269.94*x*+148.58	0.9993	0.25-	7.5	0.05	0.17
4-MBC	*y*=5470.83*x*+384.16	0.9993	0.25-	7.5	0.06	0.18
ED-PABA	*y*=17738.58*x*-952.17	0.9981	0.25-	7.5	0.04	0.12
EHMC	*y*=18832.71*x*+271.52	0.9987	0.25-	7.5	0.07	0.22
CBM	*y*=2603.48*x*-380.52	0.9989	1.25-	37.5	0.26	0.87
OC	*y*=12870.92*x*-327.76	0.9984	1.25-	37.5	0.18	0.60
BMDBM	*y*=5092.99*x*-482.54	0.9998	2.5-	75	0.63	2.10
DT	*y*=4859.05*x*-609.55	0.9994	1.25-	37.5	0.16	0.53
DHHB	*y*=5417.59*x*-786.16	0.9989	2.5-	75	0.40	1.33

* *y*: peak area; *x*: mass concentration, mg/L.

### 2.4 回收率与精密度

常用的防晒类化妆品基质为霜类和乳类,因此分别取不含目标物的霜类和乳类空白样品,在3个水平下进行加标回收试验,每个水平平行测定6次,结果见表4。结果显示13种防晒剂在霜类基质中的加标回收率为88.7%~103.6%, RSD为1.7%~4.9%,在乳类基质中的加标回收率为88.4%~102.3%, RSD为1.2%~3.9%,表明本方法的重复性和稳定性良好。

**表 4:** 13种防晒剂的加标回收率和相对标准偏差(*n*=6)

Compound	Spiked/ (mg/g)	Cream			Lotion	
		Recovery/ %	RSD/ %		Recovery/ %	RSD/ %
ES	1.25	94.5	1.9		94.2	2.5
	5	96.7	3.7		97.4	2.4
	10	101.6	2.1		100.3	1.6
HMS	2.5	95.1	2.7		92.5	2.8
	10	91.4	2.7		95.6	3.6
	25	103.6	2.2		102.3	1.7
3-BC	2.5	95.9	3.3		93.7	3.0
	10	94.4	2.4		94.1	2.8
	25	94.8	3.4		92.7	1.2
BP-3	2.5	89.8	3.1		90.8	2.7
	10	90.7	2.6		91.9	2.3
	25	90.8	3.2		89.8	1.7
IMC	2.5	92.5	2.9		88.4	3.6
	10	89.7	2.9		93.6	2.1
	25	91.1	3.0		89.8	1.8
4-MBC	2.5	88.7	3.8		91.4	2.6
	10	95.9	1.7		98.9	1.6
	25	93.7	2.7		92.4	1.6
ED-PABA	2.5	91.5	3.7		92.7	3.9
	10	91.8	3.8		90.1	3.7
	25	95.0	2.8		92.4	2.2
EHMC	2.5	90.3	2.6		89.4	2.7
	10	89.7	2.7		89.7	2.7
	25	92.9	2.4		95.0	1.9
CBM	10	92.8	4.9		94.1	2.4
	40	89.7	2.6		89.4	3.1
	100	92.6	3.4		94.0	1.7
OC	10	89.5	4.7		89.3	2.6
	40	95.8	2.3		94.3	3.8
	100	93.2	3.0		92.6	1.2
BMDBM	25	94.7	3.5		90.3	2.7
	100	93.5	2.2		96.5	2.7
	250	95.3	2.6		94.7	1.8
DT	10	93.7	3.6		94.6	3.1
	40	94.8	2.9		95.2	3.0
	100	94.8	3.5		93.2	2.5
DHHB	25	95.7	2.4		91.7	2.8
	100	93.9	3.0		96.3	2.2
	250	95.6	1.8		95.3	1.7

### 2.5 样品结果分析

随着防晒剂的广泛使用,防晒剂不仅用于防晒类化妆品中,在其他种类的化妆品中也常有添加,而这类产品属于日常监管的盲区,其防晒剂的添加水平并不清楚。美白类化妆品由于使用普遍,其产品中常添加防晒剂成分,依据本文方法测定了5批含有防晒剂的美白类化妆品中的防晒剂含量,结果见表5。实验结果显示,检测出的5种防晒剂含量为0.8%~5.2%,符合相关规定。

**表 5:** 样品的测定结果

Compound	Contents/%				
	Cream 1	Cream 2	Lotion 1	Lotion 2	Lotion 3
4-MBC	-	2.6	1.6	-	-
EHMC	4.1	3.6	3.1	4.3	5.2
OC	-	3.6	-	-	-
BMDBM	-	1.5	-	-	-
DHHB	-	-	0.8	-	-

-: not detected or less than limit of detection.

## 3 结论

本工作建立了同时测定化妆品中13种防晒剂的气相色谱-质谱方法。该方法定性定量准确,适用于一般基质的化妆品中防晒剂的测定,操作简单,安全度高,精密度高,检出限低,满足化妆品的检测要求,为市场监管和实验室检测提供了新的技术支持。测定了5批美白类化妆品中5种防晒剂的含量,结果符合相关规定。
